# Atmospheric water harvesting using functionalized carbon nanocones

**DOI:** 10.3762/bjnano.14.1

**Published:** 2023-01-02

**Authors:** Fernanda R Leivas, Marcia C Barbosa

**Affiliations:** 1 Instituto de Física, Universidade Federal do Rio Grande do Sul, CP 15051, 91501-970, Porto Alegre, RS, Brazil

**Keywords:** atmospheric water harvesting, hydrophilicity, hydrophobicity, nanocones, nanotechnology

## Abstract

In this work, we propose a method to harvest liquid water from water vapor using carbon nanocones. The condensation occurs due to the presence of hydrophilic sites at the nanocone entrance. The functionalization, together with the high mobility of water inside nanostructures, leads to a fast water flow through the nanostructure. We show using molecular dynamics simulations that this device is able to collect water if the surface functionalization is properly selected.

## Introduction

Despite water being abundant on Earth, there are at least four billion people suffering from water scarcity [1]. The lack of potable water results from a number of factors such as monoculture, increasing deforestation, and a growing population [2–5]. In order to circumvent the problem of lack of fresh water, scientists are developing alternative processes such as filtration of contaminated water [6], desalinization [7], and the collection of water from the atmosphere [8]. Atmospheric water harvesting (AWH) is an interesting option to obtain fresh water, particularly in arid and semi-arid areas, where other sources of water are inaccessible, and populations have long been suffering from water scarcity [9]. There are different processes to develop AWH such as condensing and collecting moisture, cooling ambient air below its dew point [10–11], and using chemical and physical processes involving absorption and adsorption [9,12–13]. Many of these mechanisms are inspired by structures found in nature (biomimetic designs), which use hierarchical nano/microstructures to collect water. Some examples are the *Trifolium pratense* plant, the *Cotula fallax* cactus, and the *Uloborus walckenaerius* spider [14–16]. Usually, these biomimetic designs have an asymmetrical shape that energetically drives the directional transport of water. The cactus for example has spikes where droplets move from the tip to the base, or from the higher to the lower Laplace area.

One mechanism developed by nature to capture liquid water from water vapor is present in the Namibian desert beetle, which collects water from morning steam in the desert [17]. This beetle has hydrophilic spots on its back, which transform vapor into liquid water. For the collection to be efficient, below the hydrophilic spots, its wings are hydrophobic, and the captured water moves from hydrophilic to hydrophobic parts driven by gravity. The efficiency of this process led to the development of mimetic strategies [18–21], which require the combination of wetting and dewetting properties used by the beetle. The hydrophobic region, as is also the case for the cactus, is fundamental for the mobility of water.

Water presents other kinds of anomalous behavior in addition to the hydrophobicity described above. The phenomena of density increasing with temperature at constant pressure and diffusion coefficients increasing with density at constant temperature were observed in experiments and simulations in bulk water [22–24]. Water presents both super flow and slowing down when confined in biological structures with the presence of hydrophobic and hydrophilic sites [25]. Water confined in hydrophobic structures, such as carbon nanotubes with diameters below 2 nm, exhibits a fast flow that exceeds values provided by classical hydrodynamics [26]. This super flow is observed because water is pushed away from the hydrophobic surface, forming a single line of molecules that moves in a stressless manner. This behavior only appears because at the nanometric scale water cannot be described as a continuous medium, and classical hydrodynamic equations fail.

Water super flow in nanostructures has been explored in processes of separating water from salt or from other contaminants. This high mobility of water under nanoconfinement requires huge pressure and, consequently, a lot of energy [27–28]. In order to help water entrance and decrease the amount of required pressure, nanotubes have been functionalized with hydrophilic groups [29–30]. The addition of hydrophilic regions in small diameter environments, however, decreases the velocity of water molecules [31].

The high flow of water in nanostructures is also useful for capturing water from the atmosphere. Nanotubes with hydrophilic sites for water capture and hydrophobic regions for the movement of water to reservoirs [32–33] have been analyzed. Despite reasonable results on the capacity of capturing water, the small diameter of the nanotube entrance requires high pressures for the water to enter, which makes the process energetically costly.

A geometry that combines a large surface for capturing water and a small radius for making water molecules flow fast is the nanocone. Carbon nanocones (CNCs), also called nanohorns are conical structures that are predominantly made of carbon, typically 2–5 nm in diameter and 40–50 nm in length. They occur on the surface of natural graphite. Void CNCs can be produced, for example, by decomposing hydrocarbons with a plasma torch [34]. Other simple techniques of production [35] and reduction [36] have also been recently developed. CNCs are completely hydrophobic, but they can be functionalized to make some parts hydrophilic while keeping the other parts hydrophobic.

The study of the behavior of water inside nanocones is relevant because of the structural advantages of nanocones [37–38]. The flow of water in nanocones is higher than the mobility observed in nanotubes [39–40]. In the presence of ions, water flows through a charged nanocone under an electrical field [41–42], and this flow is higher than the one induced by pressure. Consequently, the desalination performance observed in carbon nanocones is better than that observed in nanotubes or nanometric monolayers of graphene and MoS_2_ [43–44]. Another advantage of the cone format is the possibility of capturing more water at the larger diameter entrance, without losing the high flow at the reduced diameter in other parts of the cone. As the example of the Namibian desert beetle shows, the introduction of hydrophilic groups at the nanocone entrance favors the condensation of water while the hydrophobic sites at the smaller side of the cone generate a fast flow. This combination of nanotube shape and functionalization is key for making a device able to capture water.

In this work, we investigate through molecular dynamics simulations the process of capturing and collecting water in a functionalized carbon nanocone. The process is analyzed in a system in which the larger diameter of the cone is in contact with a vapor reservoir and the smaller diameter is in contact with an initially empty reservoir. The nanocone has hydrophilic and hydrophobic regions, the combination of which generates a fast flow without the need of imposing pressure to the system. Note that unlike the case of cactus spines, we present a model in which water is driven from the base (larger area) to the tip of the canonical structure by wettability variations combined with a nanometric design to improve water flow [39–40].

The paper is organized as follows: In the section “Model and Simulation Details”, the model is presented and the simulation method is explained. In the “Results and Discussion” section, the simulation results are shown and the water harvesting by the nanocone for different hydrophilic interactions is evaluated. Section “Conclusion” summarize the results.

## Model and Simulation Details

**Figure 1:**
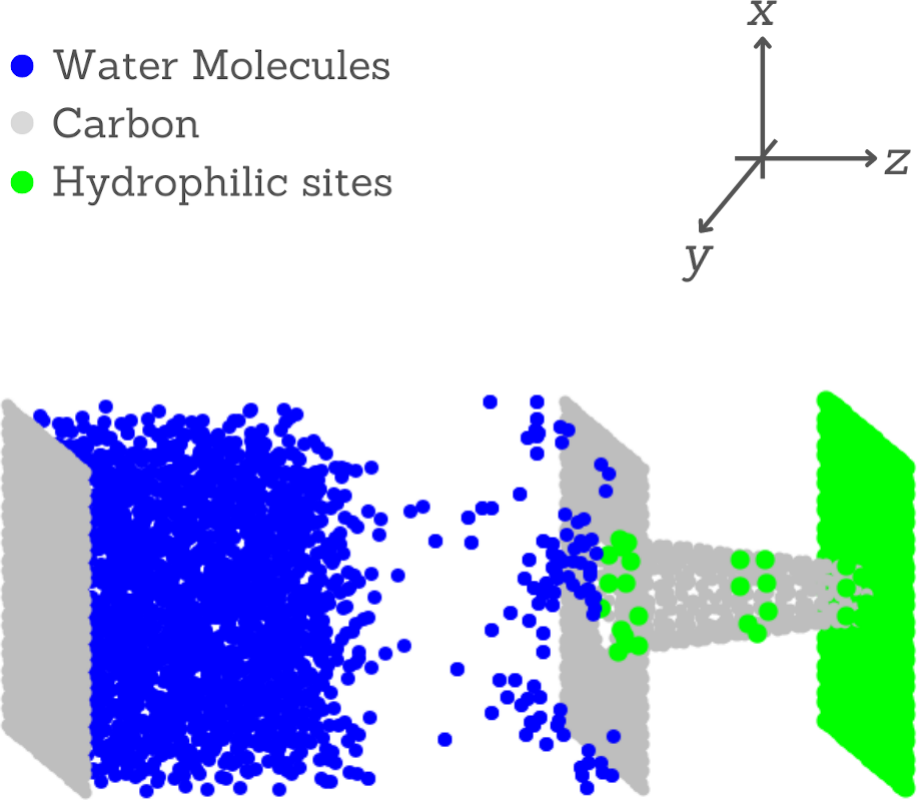
A snapshot of the simulation system. A liquid–vapor reservoir in contact with a carbon slab and with the nanocone hydrophilic base. The nanocone tip is in contact with the hydrophilic slab of a collecting reservoir.

The system is illustrated in Figure 1. It is composed of a conical carbon nanochannel between two slabs with a length of 50 Å. One slab represents hydrophilic atoms (green), and the other slab represents hydrophobic carbon atoms (gray). Both slabs are coupled to a reservoir. The hydrophobic slab is connected to a water vapor reservoir while the hydrophilic slab is connected initially to a vacuum reservoir. All slabs are maintained rigid during the simulation.

The system shown in Figure 1 is made of a reservoir of size 50 × 54 × 50 Å^3^. This reservoir has two regions, namely a liquid water region on the left and a water vapor region on the right. The number of water molecules in this simulation is 1473. The density of the vapor system is 0.38 g/cm^3^. As the simulation is conducted using an NVT ensemble, the pressure is variable.

**Figure 2:**
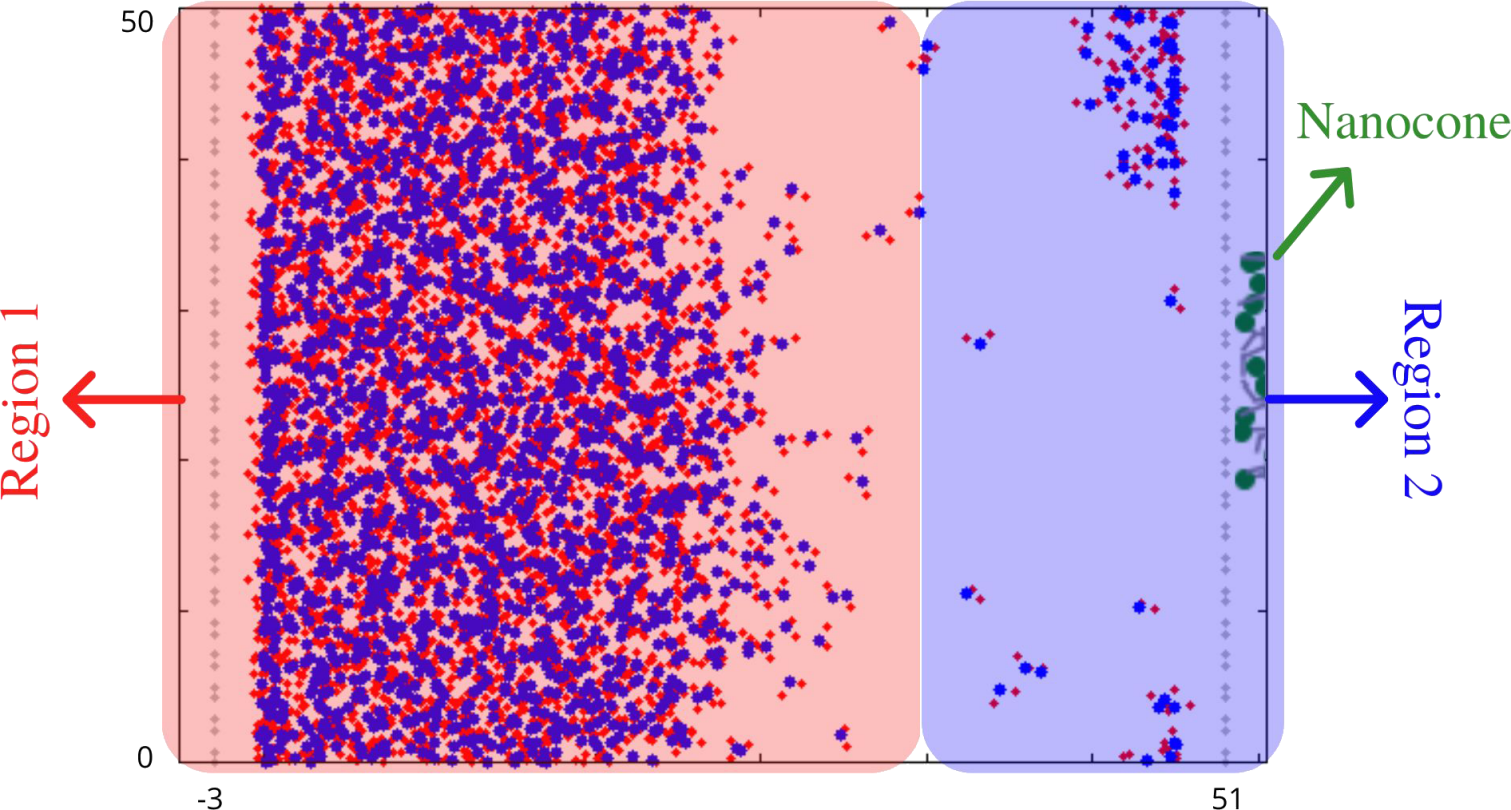
Part of the simulation box illustrating the two types of thermal control used in the simulation. The red area represents region 1 with a temperature dynamically changing between 800 and 300 K while the blue area is region 2 with a fixed temperature of 300 K.

The condensation is produced by a combination of thermostats, as illustrated in Figure 2, where the liquid region is illustrated in red while the vapor region is shown in blue. The red region does not have fixed thermostat, but the temperature varies from 800 to 300 K in a dynamic process every 10000 temporal steps. The blue region, thermostat 2, maintains a constant temperature of 300 K during the entire simulation. The variation of temperature in thermostat 1 is responsible for the condensation at reservoir 2. The idea of combining thermostats to produce vapor is not new. It has already been used to reproduce water evaporation and condensation [45–46].

**Figure 3:**
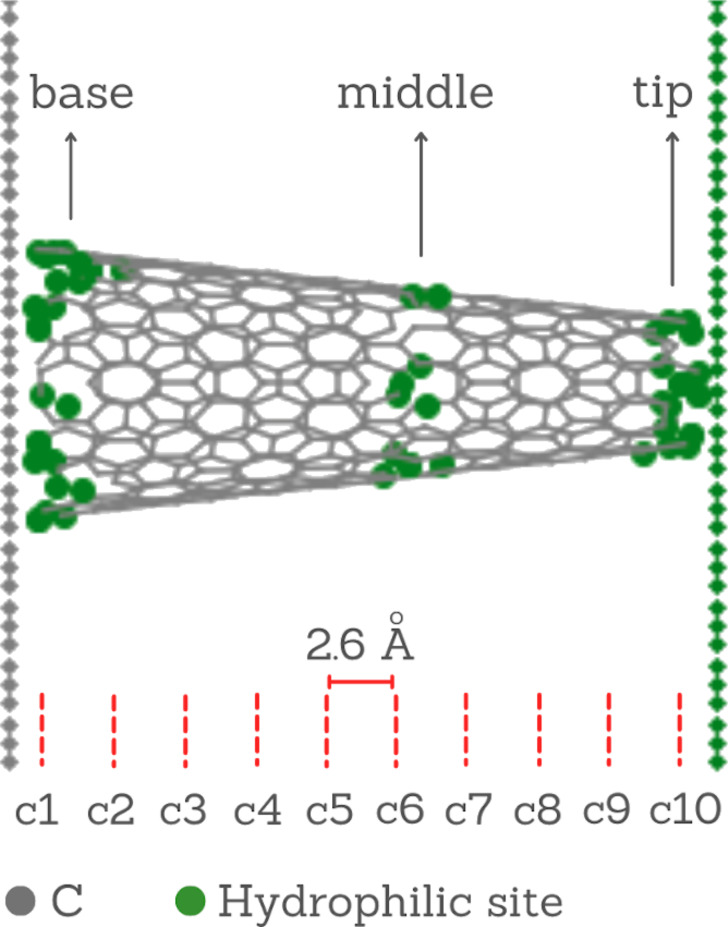
Carbon nanocone with 26 Å length, 8.2 Å tip diameter, and 17 Å of base diameter. Hydrophilic rings are present at the base, the tip, and the middle of the nanocone.

In contact with the carbon slab on region 1, there is a carbon nanocone constructed by cutting the apex angle, as illustrated in Figure 3. This nanocone has a length of 26 Å. The smaller pore at the tip has a diameter of 8.2 Å, and the larger pore at the base has a diameter of 17Å. Along the CNC, there are three ring-shaped regions with hydrophilic sites at the base, at the middle, and at the tip. The hydrophilic rings are modeled as effective water-wall potentials ε_r_. The CNC and the sheets were held fixed during the simulations, and water molecules inside the nanocone were treated with a thermostat at 300 K.

Carbon nanocones can be produced with five different apex angles [47]. Here we use the aperture of 19.2° since it is easier to produce at large scale [34]. Also, it is the nanocone that achieves the highest values of water flux compared with the other apex angles. It also presents a lower energy barrier when compared with carbon nanotubes [39].

The smaller side of the nanocone ends in a hydrophilic surface, which has the same structure as the hydrophobic slab. This hydrophilic surface forms the collector reservoir, in which there is no water at the beginning of the simulation. The dimensions of this reservoir are 50 × 20 × 50 Å^3^. The water molecules collected by this reservoir are maintained at 300 K in a thermostat as in region 2 (Figure 2).

Molecular dynamics simulations were performed using the LAMMPS [48] package using an NVT ensemble with a timestep of 0.1 fs. The TIP4P/2005 [49] water model was used since it provides a satisfactory description of self-diffusion coefficient [50], phase diagram, vapor–liquid equilibria [51–52], vapor pressure, and critical temperature, despite being a simple model [15,53].

The SHAKE algorithm was employed to keep the rigidity of water molecules. The oxygen–carbon Lennard-Jones (LJ) pair-wise non-bonded interaction, ε_O−C_ = 0.126 kcal/mol and σ_O−C_ = 3.279 Å, was calculated using the Lorentz–Berthelot mixing rules [54]. For the interaction between hydrophilic sites and water, the same σ of oxygen–carbon interaction was fixed (σ_O−HS_ = σ_O−C_), but the potential well ε_O−HS_ = ε*_i_* was varied. The LJ cutoff distance was 12 Å, and the long-range electrostatic interaction was treated by the particle–particle mesh method. Periodic boundary conditions were applied along the *x*- and *y*-directions, and non-periodic boundary conditions were applied along the *z*-direction (see Figure 1).

## Results and Discussion

Figure 1 illustrates the analyzed system, composed of a water vapor reservoir in contact with the base of the nanocone. If the nanocone is fully hydrophobic, no water crosses the nanocone. Therefore, hydrophilic rings are necessary for the water to enter and flow through the nanocone. We employ the Lennard-Jones potential to calculate the interaction between the nanocone wall and water. Within this approach, the variable that determines the wettability of the surface is the potential well defined by ε_r_. The higher the value of ε_r_, the more hydrophilic the surface is. Here we employed 1.5 ≥ ε_r_ ≥ 0.8.

**Figure 4:**
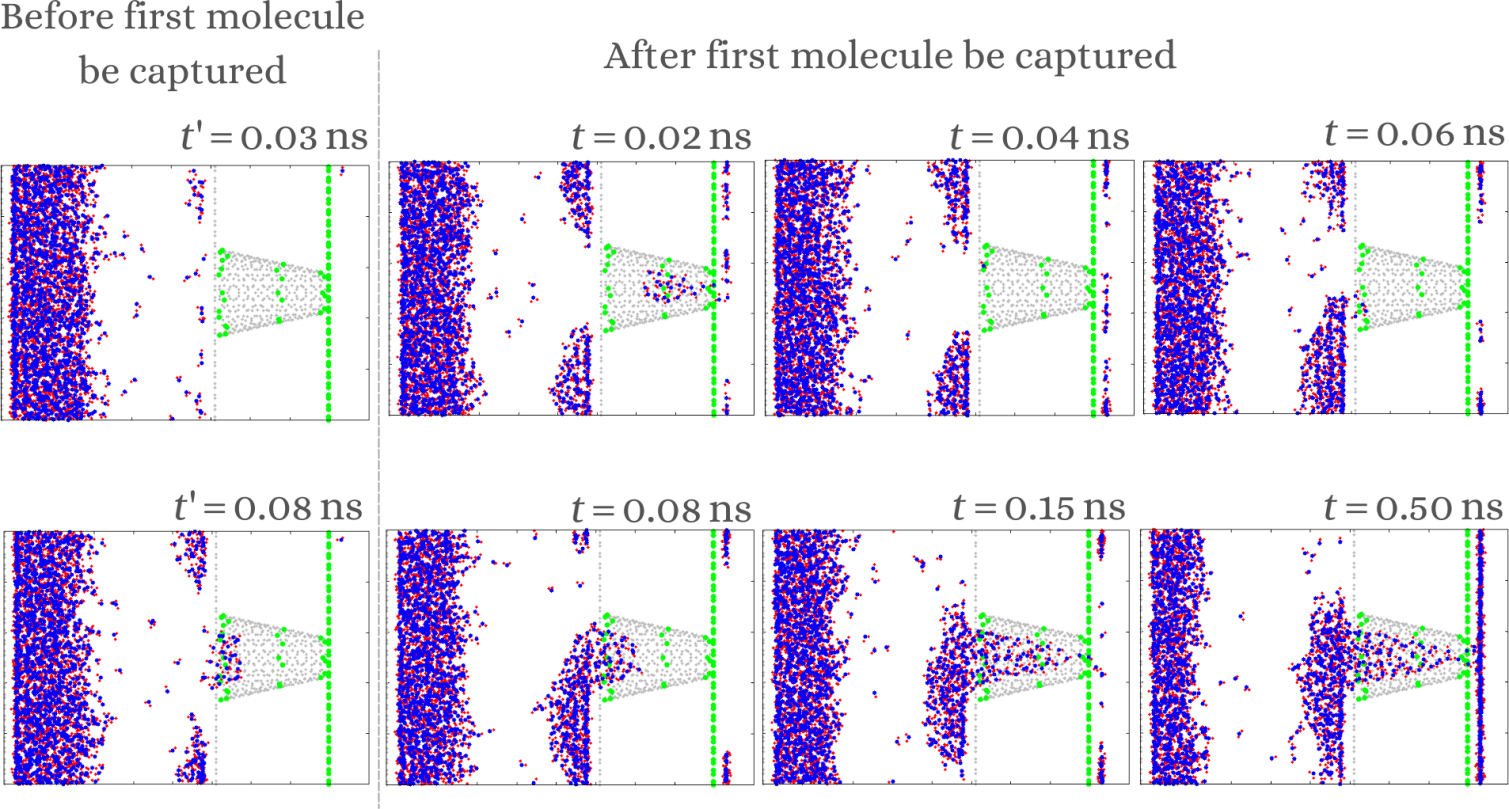
Snapshots of the temporal evolution of the vapor system using ε*_i_* = 1.1.

The water harvesting has been modeled according to the following mechanism: First, the vapor generated in region 1 from Figure 2 condenses into the slab of region 2, forming droplets. These droplets are attracted to the hydrophilic sites of the nanocone, as shown in Figure 4. Eventually, the droplets are moved to the middle and, then, to the tip of the nanocone due to the combination of hydrophilic and hydrophobic sites. Without them, the droplet is stuck at the base of the nanochannel. After an initial period (see *t* = 0.15 ns in Figure 4), only a large droplet remains being absorbed by the nanocone. This drop is fed by the vapor, forming a continuous flow of molecules that reach from the base to the tip of the nanocone, crossing to the collecting reservoir. This process stops once the collecting slab is filled. The time it takes for this to happen, after the first molecule is captured, depends on the initial conditions and ranges from 0.3 to 0.5 ns.

**Figure 5:**
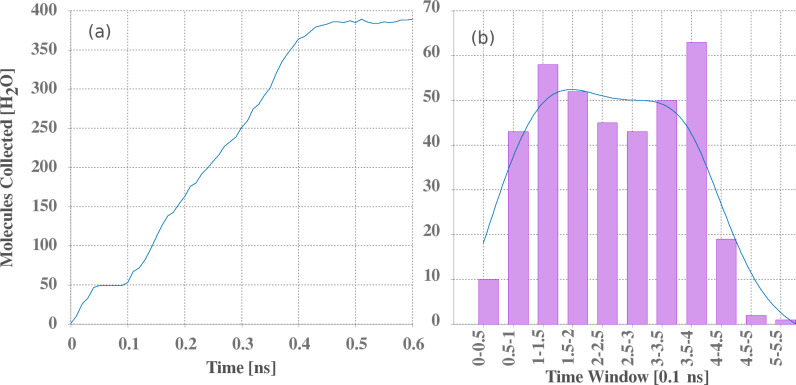
(a) Number and (b) histogram of collected water molecules as functions of (a) the time and (b) time intervals.

The number of collected molecules and the histogram versus time for the vapor reservoir system are presented in Figure 5a and in Figure 5b, respectively. Both graphs were obtained for one sample. The water harvesting (time interval 0.1–0.4 ns) exhibits a linear growth, called here “linear regime”. This regime is achieved when a large droplet is formed at the base of the nanocone as described above, enters the nanocone, and undergoes cohesive dynamics.

**Figure 6:**
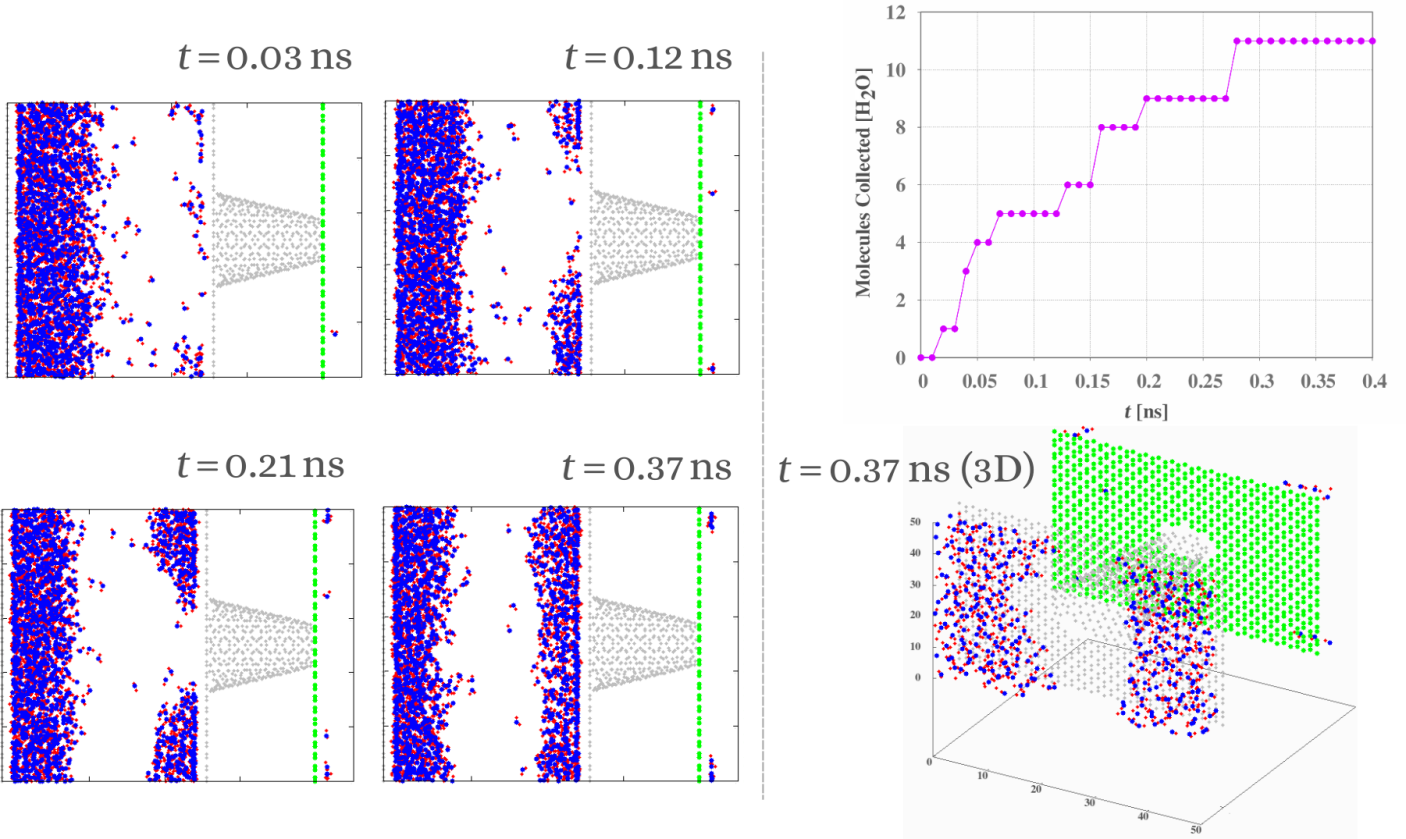
2D snapshots of the temporal evolution for vapor for a hydrophobic nanocone (left), the number of collected water molecules as a function of the time (top right), and a 3D snapshot for *t* = 0.37 ns (bottom right).

In order to characterize and understand the impact of the hydrophilic rings along the nanocone, we also present in Figure 6 snapshots of the temporal evolution and the number of water molecules collected over time by a completely hydrophobic nanocone. In the presence of hydrophilic rings, the nanocone captured more than 350 molecules in 0.4 ns. During the same period of time, the hydrophobic nanocone captured 11 molecules (less than 10%). For this simulation, we did not change the wettability of the hydrophilic slab (ε_r_ = 1.1). Without that, no molecule would have been captured. In the case of the completely hydrophobic nanocone, no droplet is formed in the base of the nanocone. Only very few molecules randomly enter and are successfully harvested, but this number is negligible. In Figure 6 (bottom right), we also present a 3D snapshot for *t* = 0.37 ns to demonstrate that there are no molecules at the entrance of the nanocone.

**Figure 7:**
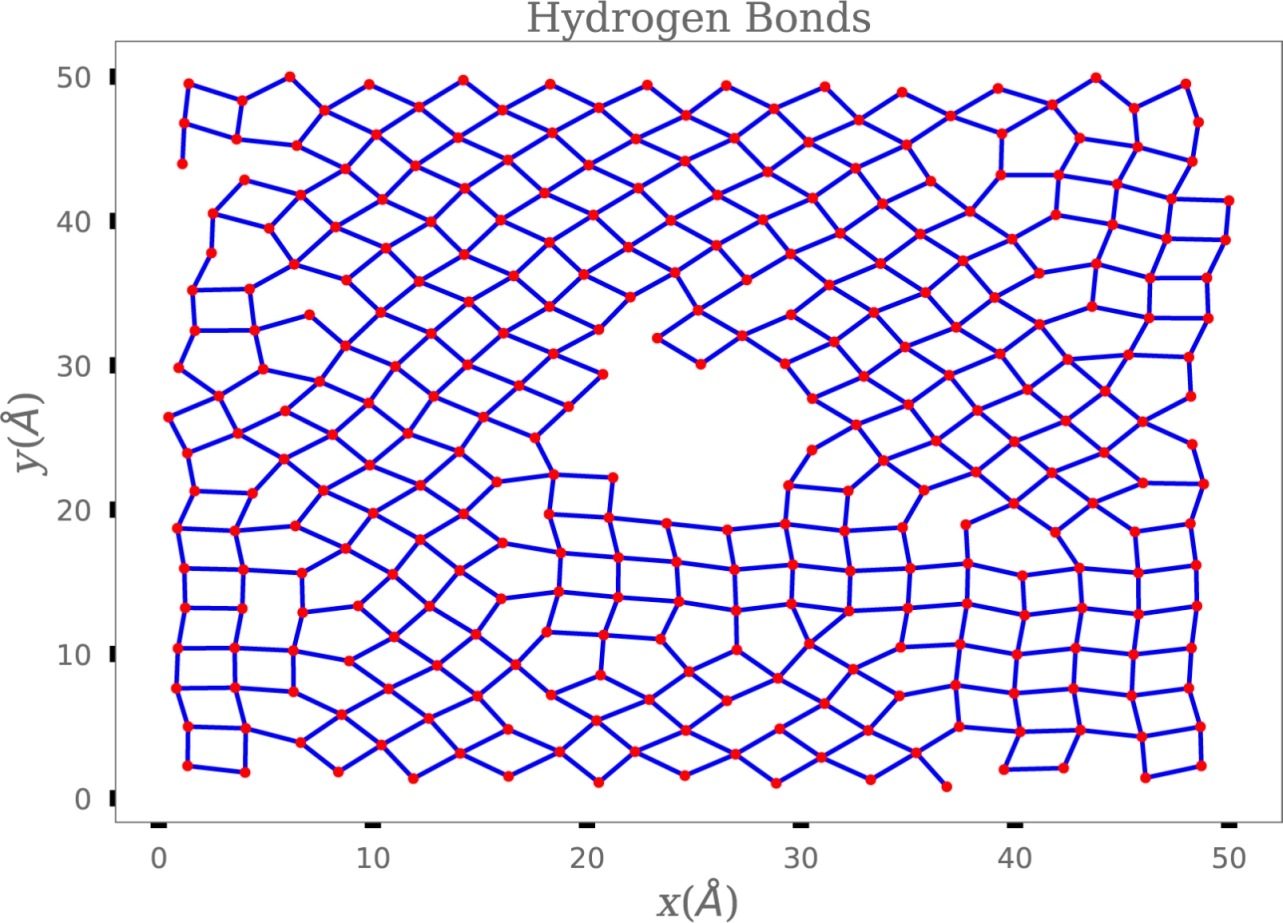
A snapshot of water molecules (red dots) on the attractive slab and hydrogen bonds (blue lines) at *t* = 0.5 ns. The central region is where the nanocone is placed; we did not plot the molecules for this region. The hydrogen bonds were calculated using the distances and angles between water molecules.

**Figure 8:**
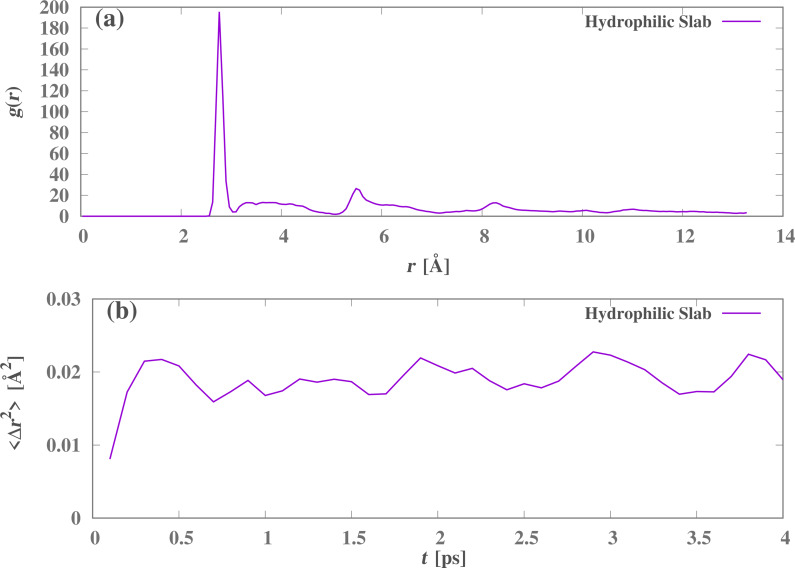
(a) Radial distribution function and (b) mean square displacement of the water molecules on the attractive slab at *t* = 0.5 ns and ε_r_ = 1.1.

For the case of the nanocone with hydrophilic rings, Figure 7 shows a snapshot of the water molecules on the hydrophilic slab of the collector reservoir, after the flow ceases and the number of water molecules in the reservoir becomes constant. Note that these molecules and their hydrogen bonds are placed in a crystal-like arrangement. Figure 8a shows the radial distribution function, which is characteristic of an ordered structure in two dimensions. Figure 8b illustrates the mean square displacement of the water molecules on the collecting slab, indicating a very small and constant mobility, thus confirming the ice-like behavior.

**Figure 9:**
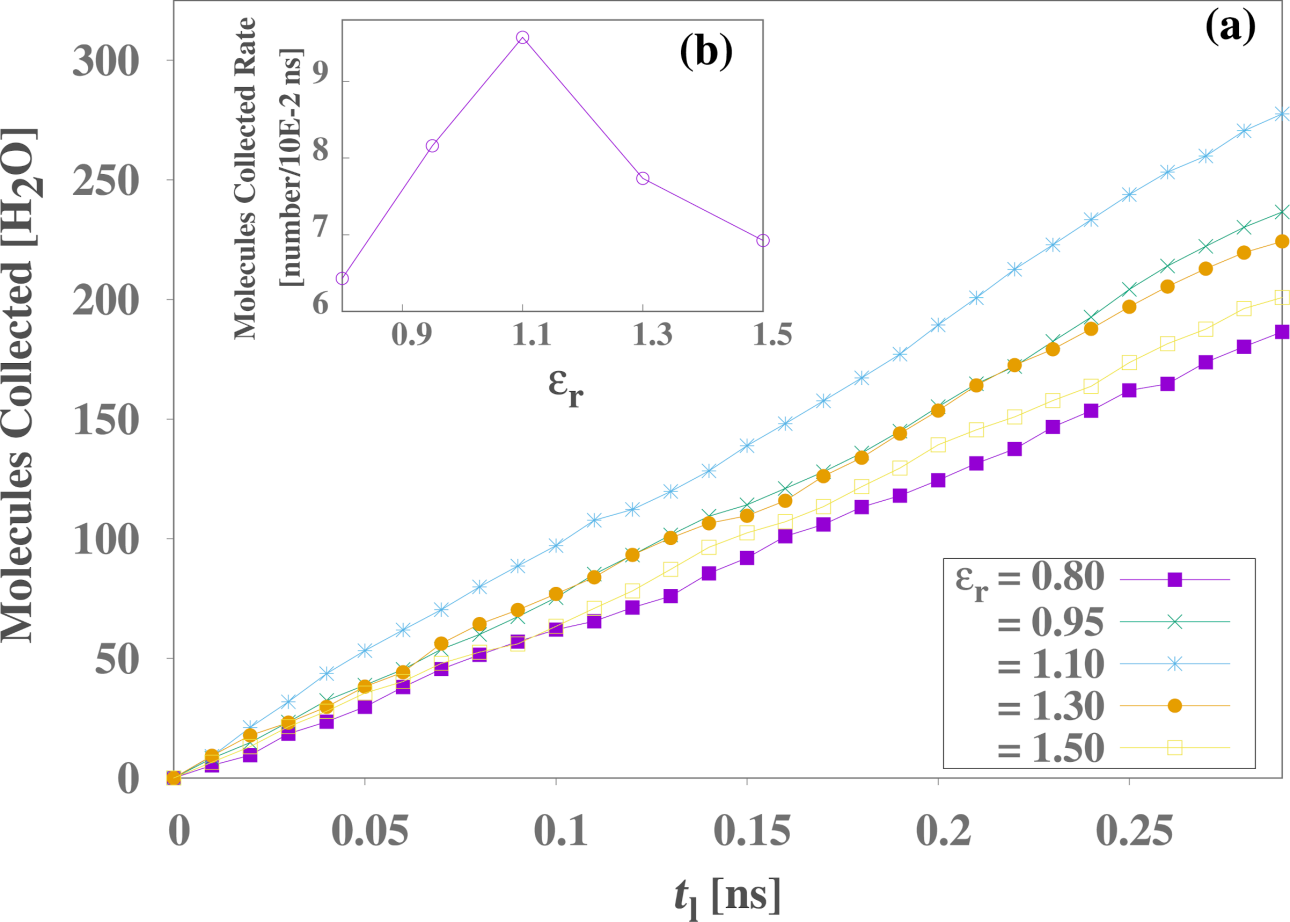
(a) Number of collected molecules as a function of the time (ns) for different ε_r_. (b) Mean collected rate (MCR) as a function of ε_r_.

What happens to the system when hydrophilic interactions between water and surfaces are increased? In order to answer this question, Figure 9 illustrates the number of collected molecules as a function of time for different values of water–wall attraction, that is, ε_r_ = 0.80, 0.95, 1.1, 1.3, and 1.5. Each line is averaged over five samples. Note that the slopes of lines for fixed ε_r_ present a non-monotonic behavior with ε_r_. In order to understand the impact of varying attraction, we calculated the mean collected rate of molecules (MCR) per unit of time (10^−2^ ns), using


[1] $$\text{MCR}=\sum_{i=0}^{i=t_{\text{tot}}}\frac{\left(Nm_{i}-Nm_{i-1}\right)}{t_{\text{tot}}}.$$

Figure 9b shows the MCR of molecules as a function of ε_r_. For values of ε_r_ below a certain threshold, the MCR increases in proportion to ε_r_. The movement of molecules in the nanocone depends on the combination of attraction on hydrophilic sites and repulsion on hydrophobic sites. Enhancing hydrophilic attraction increases the number of water molecules attracted to the base of the nanocone. For ε_r_ values above a certain threshold, however, the MCR decreases. In this case, the hydrophilic interaction with the rings is too strong, and water molecules tend to be stuck at the rings. The maximum rate occurs for ε_r_ ≈ 1.1. This result for the optimum interaction between the oxygen and the nanocone atoms might be helpful to select potential functional groups to be added to the carbon nanocone.

In order to understand how ε_r_ impacts the water movement, we computed the flow. As the diameter of a conical object varies with length, the axial flux of molecules also varies from point to point in the cone. Therefore, we selected ten regions equally spaced along the nanocone, as shown in Figure 1, and we calculated the flux at each segment using the expression


[2] $$J_{i}=\frac{n_{\text{ltr}}-n_{\text{rtl}}}{A_{i}N_{\text{steps}}\delta t},$$

where *n*_ltr_ is the number of molecules that cross a region of the nanotube from left to right, and *n*_rtl_ is the number of molecules that cross from right to left. 
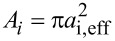
 is the area of region *i* with radius *a_i_*, and *a*_i,eff_ = *a_i_* − σ/2 is the effective radius available for water σ = 3.1589. *N*_steps_ = 10^4^ is the total number of steps used to calculate the flux, and δ*t* = 0.1 fs is the time step.

**Figure 10:**
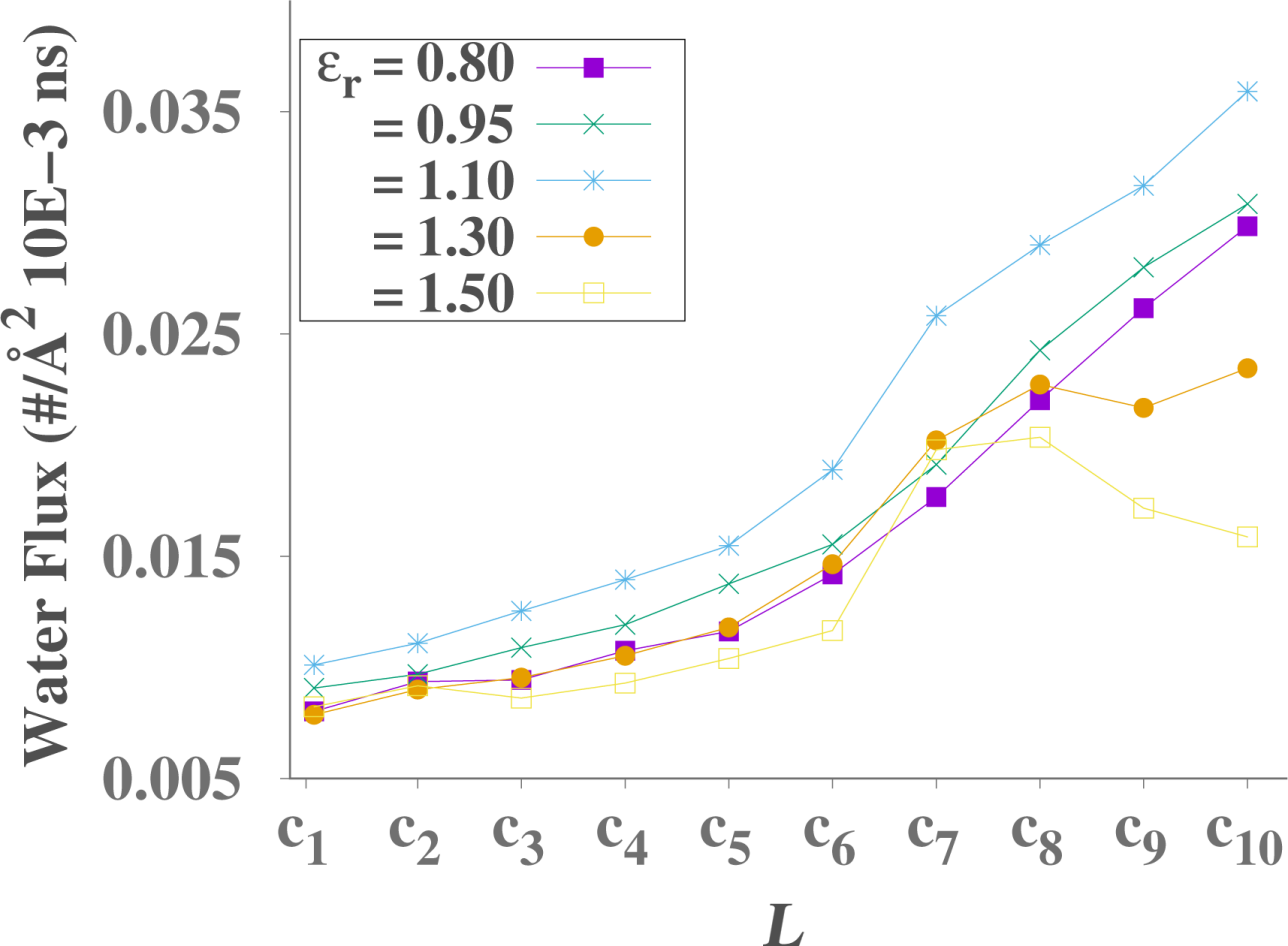
Graph of water flux in different regions of the nanocone (Figure 3), for different values of the potential well ε_r_.

Figure 10 shows the flux *J_i_* as a function of the region (length) *c_i_* (Figure 3) of the nanocone for different values of attraction ε_r_. Each value was averaged over five samples with 8 × 10^5^ fs. This graph confirms the behavior observed in Figure 9. The increase of water mobility with the increase of ε_r_ up to ε*_r_* = 1.1, and the decrease of *J_i_* for ε*_r_* *>* 1.1. In addition, Figure 10 shows the increase in *J_i_* with the decrease in diameter for the lower values of hydrophobicity, ε*_r_* = 0.80, 0.95, and 1.1.

When ε_r_ *>* 1.1, a non-monotonic behavior is observed. The decrease in flux with the decrease in diameter for *c*_8_, *c*_9_, and *c*_10_ and ε_r_ = 1.30 and 1.50 is a consequence of the strong interaction between water molecules and the hydrophilic ring in the middle of the nanocone. The increase in flux as the radius decreases is a behavior also observed in carbon nanotubes [55]. The increase in flux, followed by a decrease, with increase in hydrophobicity was also observed regarding the transport properties of nanotubes with tunable hydrophilic sites [56–57].

## Conclusion

Molecular dynamics simulations were performed to study water harvesting, using a combination of hydrophobic and hydrophilic sites in carbon nanocones in contact with a vapor water reservoir. The nanocone was constructed modeling three ring-shaped hydrophilic regions. Different from simulations and experiments with water flow in nanotubes, no external pressure was applied.

First, we observed that without the hydrophilic slab, no water molecule passes through the hydrophobic nanocone. In the presence of the hydrophilic slab at the end of a completely hydrophobic nanocone, isolated water molecules pass through, but no droplet is formed.

Then, hydrophilic rings were introduced in the nanocone, and different hydrophilic strengths of the rings were explored. Water dynamics in this case is governed by the formation of droplets outside the nanocone, and a combination of regimes forms. At the beginning, droplets condense on the slab surface. Then, these droplets are attracted by the hydrophilic base of the nanocone. They form a larger drop, which enters into the cone, thus generating a steady flux to the nanocone tip and reaching the collecting slab. This flow is generated by the combination of hydrophilic and hydrophobic sites.

The flow only stops when the collecting slab becomes filled. This slab is hydrophilic and attracts water molecules. These molecules form an ordered structure on the slab that freezes the water once the hydrophilic slab is completely filled. So, the flow is interrupted even when the collector is kept at a temperature of 300 K. A solution to keep the flow of water would be to continuously remove water molecules from the collecting slab.

The strength ε_r_ of the hydrophilic sites affects water collection and water mobility in two ways. Increasing ε_r_ pushes more droplets to the nanocone. However, if ε_r_ is too large, water molecules become trapped at the hydrophilic regions, decreasing the water mobility.

Thus, the nanocone can be suggested as an alternative to collect water from vapor without the use of high pressure, provided that it combines hydrophobic and hydrophilic regions with an optimized value of ε_r_.
